# Study of the prevalence of impaired hearing and its determinants in the city of Itajaí, Santa Catarina State, Brazil

**DOI:** 10.1590/S1808-86942012000200006

**Published:** 2015-10-20

**Authors:** Lys Maria Allenstein Gondim, Sheila Andreoli Balen, Karla Jean Zimmermann, Débora Frizzo Pagnossin, Indiara de Mesquita Fialho, Simone Mariotto Roggia

**Affiliations:** a Specialized medical doctor in otorhinolaryngology (Itajaí/SC) – Curumim Clinic. Vale do Itajaí University (UNIVALI). Hearing Health Care Unit & Pequeno Anjo Child University Hospital); b Doctoral degree (Professor of the Speech Therapy Course, Rio Grande do Norte Federal University (UFRN), Natal/RN); c Master's degree (Professor of the Speech Therapy Course & Hearing Health Care Unit, UNIVALI, Itajaí/SC); d Master's degree (Professor of the Speech Therapy Course. Coordinator of the Hearing Health Care Unit, UNIVALI, Itajaí/SC); e Doctoral degree (Professor of the Speech Therapy Course, Santa Catarina Federal University (UFSC), Florianópolis, SC). Vale do Itajai University – Universidade do Vale do Itajai (UNIVALI), Itajaí/SC

**Keywords:** brazil, deafness, hearing loss, prevalence

## Abstract

The number of people with impaired hearing is increasing; knowing its magnitude is essential for public health.

**Objective:**

To study the prevalence and determinants of impaired hearing in Itajaí/SC.

**Methods:**

A population-based survey based on a World Health Organization protocol. Field research was carried out from July 2008 to 2011. Procedures for evaluating hearing in households: questionnaire, measurement of noise, otoscopy, pure tone audiometry at 1000, 2000, and 4000 Hz, tympanometry, and acoustic reflexes: individuals above 4 years; children under 4 years: evoked otoacoustic emissions (OAE), cocleo-palpebral reflex(CPR), tympanometry, and acoustic reflexes. In the elderly population a questionnaire of perception of hearing loss was applied.

**Results:**

The study sample consisted of 379 individuals – 45.38% were males and 54.62% were females. Age-range: 11.34% up to 10 years; 64.39% 10 to 60 years, 24.27% over 60 years. Evaluation of the best hearing ear showed that 74.1% of residents had normal hearing, 18.9% had mild hearing loss, 5.1% had moderate hearing loss, 1.9% had severe hearing loss. Disabling impaired hearing was detected in 26 subjects: one child (otitis media); four adults (one otitis, one noise-induced, two idiopathic); 21 elderlies (presbyacusis). Of eight children under 4 years all presented CPR, three were normal examinations, two had absent OAE bilaterally, one had absent OAE in the right ear and one in the left ear.

**Conclusion:**

The prevalence of disabling impaired hearing in Itajaí was 7%; the highest prevalence was in the 50-year and above age group – the main cause was presbyacusis.

## INTRODUCTION

Hearing is one of the essential senses for human communication; any abnormality in the hearing system at any stage in life may compromise the communication process.

Studies in the 1990s have shown that about 70 million people worldwide have hearing loss over 55 decibels (dB)^1^. According to the World Health Organization (WHO), there were 278 million people worldwide with hearing loss in 2005; the prevalence of incapacitating hearing loss ranged from 2.1% to 8.8% in developing countries^2^. These numbers have risen every year because of increased life expectancy in all countries. Thus, it is essential to learn about how hearing loss arises, what is its magnitude, determining factors, and prevalence in different regions and age groups for the purpose of public health management.

Although specific statistical data on hearing loss are sparse in Brazil, the Support Group for Universal Neonatal Hearing Screening (Grupo de Apoio à Triagem Auditiva Neonatal Universal or GATANU) has published data on its website suggesting that the occurrence of hearing loss in neonates is 30 for every 10,000 live births in Brazil^3^. WHO data indicates that 1.5% of the Brazilian population has some degree of hearing loss. The Brazilian demographic census in 2000 (Brazilian Geography and Statistics Institute or IBGE) showed that 3.4% of the 14.5% of the entire population that had some degree of hearing loss reported moderate to severe difficulty in hearing^4^.

A review of several Brazilian epidemiologic studies on audiology that was published in 2011^5^ concluded that there is increasing concern about work-related hearing loss. Workers exposed to occupational noise have received more attention from researchers in epidemiology compared to other groups, such as the neonatal and elderly populations, about which research has been sparse. This review also underlined the need for further studies on hearing loss to support healthcare planning adapted to the needs of each region, to reduce public health costs, and to improve the quality of life of the population.

Several controversies remain about the incidence and prevalence of hearing loss. Definitions and criteria are still heterogeneous in research studies and demographic and epidemiologic data on hearing loss in Brazil remain scarce.

Because of the geographical extent of Brazil and population differences among regions, further studies using a common methodology are needed.

At present two Brazilian population studies using a method proposed by the WHO have been conducted. Béria et al.^6^ undertook the first of these studies in Canoas, RS, showing that 26.1% of that population had some degree of hearing loss; 19.3% of these had mild hearing loss, and 6.8% had incapacitating hearing loss (5.4% had moderate hearing loss, 1.2% had severe hearing loss, and 0.2% had profound hearing loss). Bevilacqua et al.^7^ undertook the second study in Monte Negro, RO, and showed that 16.5% of the population had some degree of hearing loss; 11.7% had mild hearing loss, and 4.8% had incapacitating hearing loss (3.7% had moderate hearing loss, 1.2% had severe hearing loss, and the percentage of profound hearing loss was 0%).

The main etiologic factors of hearing loss in infancy were genetic and neonatal causes, such as premature birth, low birth-weight, anoxia, hyperbilirubinemia, ototoxic drugs, sequelae and/or complications of diseases (otitis media, rubella, rubeola, and meningitis). The most common causes in adults are presbycusis, followed by noise-induced hearing loss^8^.

Because the majority of hearing losses can be avoided, or preventive measures can minimize the difficulties that result from hearing loss, an early diagnosis and intervention, as well as knowledge about the magnitude of hearing, are paramount.

Therefore, the main purpose of this study was to investigate the prevalence of hearing loss and its determinants in the city of Itajaí, SC, by applying a population-based study according to a WHO protocol to generate a standardized database containing epidemiologic information about hearing loss in Brazil.

## METHODS

The institutional review board of Vale do Itajai University (UNIVALI) approved this study (no. 153/2008).

The population of Itajaí is 183,373 people (IBGE, 2011^9^), distributed in 141 census sectors. A cross-sectional contemporary cohort study based on cluster sampling of the population, as proposed in a WHO^10^ protocol, was proposed. The term “cluster” implies a set of units that form a natural group for sampling (generally households). Households in each cluster were randomly chosen for visits based on the IBGE reference for the mean number of people per household in Itajaí (3.16 inhabitants). Ten percent of the census sectors of Itajaí were chosen randomly (14 sectors out of 141 in the municipality) to set the number of individuals to be evaluated per census sector; the calculated sample was divided by the number of chosen sectors. The software EPI-INFO 6 was used for calculating the sample, based on a 10% estimated prevalence of hearing loss (suggested by a WHO/1999 protocol); the probability of error was 3%, of accuracy it was 1.4%, the confidence interval and design effect were 2.0, and the expected losses in the sample were set at 30%. Loss was considered as taking place when a household dweller was not found for examination in at least three visits of the team, or if they refused to participate. Abandoned or uninhabited houses were considered as lost, and were replaced by the neighboring households. The calculation yielded a sample comprising 421 people.

The field survey was done from July 2008 to July 2011; there were two survey teams in the field from July to November 2008, one in each shift. There was a single survey team working in alternated shifts from November to July 2011. After the random selection, sectors were ordered numerically from the lowest to the highest number to define the visiting order for the survey team. Next, a block and a corner in each sector were randomly chosen. From this corner, the fourth, eighth, twelfth houses, and so on, were visited until reaching the preestablished number of houses, visiting from the lowest to the highest number on the left side of the street. It the block ended before reaching the required number of houses for visitation, the surveyors crossed the road and recommenced on the right side, randomly choosing four in four houses.

All individuals in the selected houses were included for visitation and assessment of hearing; persons with mental deficiencies or unable to provide consent for participating in the study, and those that refused to participate after three attempts were excluded. Collective households, stores, and uninhabited houses were also excluded.

The survey team chose a few approaches to increase the receptiveness of the inhabitants of Itajaí to visitation and to provide prior information about the objectives and goals of the study. Thus, after the random selection of households, introduction letters with information about the project were delivered to all houses one week in advance. At all times team members wore an identification badge and a project T-shirt.

Participants signed a free informed consent form that had been approved by the institutional review board of Vale do Itajai University (UNIVALI); it contained information about the objectives, benefits, and risks of the study.

Visits were made to 774 households, 684 of which were privately-owned houses, 10 were collective households, and 80 were non-residential houses. Evaluations were made of 715 people; however, 336 were excluded by being part of incomplete households according to the population base research method, as suggested in WHO inclusion criteria. Thus, the sample consisted of 379 people from complete households who were evaluated; the loss was 10% of the ideal calculated sample (42 of 421 individuals).

The following procedures, based on a prevalence assessment protocol recommended by the WHO (1999), were applied: a questionnaire, measurement of environmental noise levels, meatoscopy/otoscopy, and investigation of auditory thresholds at 1000, 2000, and 4000 Hz. Transient evoked otoacoustic emissions and a behavioral audiological assessment were made of children aged 0 to 4 years. Additionally, automatic tympanometry and investigation of ipsilateral acoustic reflexes were carried out in all subjects. A questionnaire based on a translation of the *Hearing Handicap Inventory for the Elderly, Screening Version,* HHIE-S^11^, ^12^, was applied to elderly patients aged over 60 years. It contains 10 closed answer questions; the maximum score is 40 points, allocated as follows: ‘yes' (4 points), ‘sometimes' (2 points) or ‘no' (0 points), where a sum of points below 8% meant no perception of *handicap*;11% to 22% meant mild to moderate perception of handicap, and over 22% meant significant or severe perception of handicap.

The first procedure was to apply a general questionnaire to gather identification data, age, gender, education level, and work of household dwellers that were chosen for this study; this was a tactic for generating interaction with the families.

A digital sound pressure level meter (*Sound Meter* 840029) was used to measure the ambient noise level to make sure that ambient noise did not exceed 40 dBH; hearing assessments were not made in acoustic booths (WHO, 1999). The sound pressure level meter was kept on throughout the procedures.

A *Heine* Halogen K180 otoscope was used for inspecting the outer ear; the goal was to check the status of the outer ear canal and the tympanic membrane in the audiological evaluations.

Auditory thresholds were investigated in all subjects aged over 4 years. A *Welch Allyn*, model AM 232 manual audiometer and TDH 39 earphones were used. Subjects were asked to raise one of their hands when hearing a sound stimulus. Presentation of stimuli started at 60 dBHL at 1000 Hz, then at 2000 and 4000 Hz, with retesting at 1000 Hz. The procedure was repeated if a difference higher than 5 dB at the1000 Hz threshold was encountered. The sloping technique was used to study auditory thresholds.

Behavioral hearing assessment was done by observing auditory reactions to speech sounds (name, simple commands, and repetition of sentences) and by testing the cochleo-palpebral reflex (CPR) with an agogo.

An *Otoport device was used for investigating transient stimulus evoked otoacoustic emissions (TOAE); the child was seated in their mother's lap*. Tested frequencies were 1000, 1500, 2000, 3000, and 4000 Hz; the pass-fail criterion was the presence of TOAE in at least three frequency bands.

A *Handtymp device was used for automatic tympanometry and testing of ipsilateral acoustic reflexes*. Because it is a quick, objective, non-invasive procedure that does not require behavioral responses from subjects, it was applied to all age groups, including children aged below 4 years.

Mean auditory thresholds at 1000, 2000, and 4000 Hz for the best ear were used to assess the degree of hearing loss. The following parameters were applied for children aged up to 15 years: 0–15 dB – normal; 16–30 dB – mild hearing loss; 31–60 dB – moderate hearing loss; 61–80 dB – severe hearing loss; and over 81 dB – profound hearing loss. The parameters for adults were: 0–25 dB – normal; 26–40 dB – mild hearing loss; 41–60 dB – moderate hearing loss; 61–80 dB – severe hearing loss; and over 81 dB – profound hearing loss.

Incapacitating hearing loss was defined as the presence of auditory thresholds equal to 41 dB or more in adults, and 31 dB or more in children below 15 years of age. The WHO definition of incapacitating hearing loss considers only permanent hearing loss as incapacitating. In our study, as in Béria et al.s^6^ paper, non-permanent hearing losses were also included – such as those caused by otitis media.

Individuals with any abnormality in our evaluations were referred to the ‘Hearing Health Care Unit' of Vale do Itajai University (UNIVALI) for complete otorhinolaryngologic and audiology assessments if needed. Subjects were given a card with the date and time of the visit, and the reasons for this visit were explained.

For quality control telephone contact was made with the dwellers of 10% of participating households to ask about their relationship with interviewers and if all the expected procedures were carried out.

The statistical study consisted of simple and relative frequency distribution analysis, crossing the data to study the variables sex, age group, and hearing level in the best ear. The variables age and sex were compared with the IBGE (2011) population based study to check the representativeness of the sample. Data are presented as frequencies and percentages (qualitative variables), and as means and standard deviations (quantitative variables).

## RESULTS

According to IBGE (2011) census data, there were 64,679 households in Itajaí. Of these, 57,815 (89.4%) were classified as private occupied households, 57,612 (89%) with census interviews and 203 (0.4%) without census interviews, 6,801 (10.5%) were private unoccupied households, and 63 (0.1%) were classified as collective households; the mean number of dwellers per household was 3,16. For this study 774 households were visited, 684 (88.37%) of which were private, as follows: 300 (38.76%) occupied (137 complete households (17.70%) and 163 (21.05%) incomplete), 384 households were not occupied, (256 (33.07%) were closed, 128 (16.54%) were vacant); 10 (1.30%) were collective households, and 80 (10.33%) were non-residential buildings. The mean number of visited dwellers per household without incapacitating hearing loss was 2.8; this mean was 2.54 for households with at least one persons presenting incapacitating hearing loss.

The distribution by census sectors showed that there were no significant differences when considering the number of complete households and the prevalence of hearing loss per sector. Mild hearing loss was found in all of the 14 census sectors that were evaluated; moderate hearing loss was found in 10 of these sectors, and severe hearing loss was encountered in six sectors.

Of the 379 inhabitants of Itajaí that comprised the study sample, 172 (45.38%) were male and 207 (54.62%) were female.

**Figure 1:**
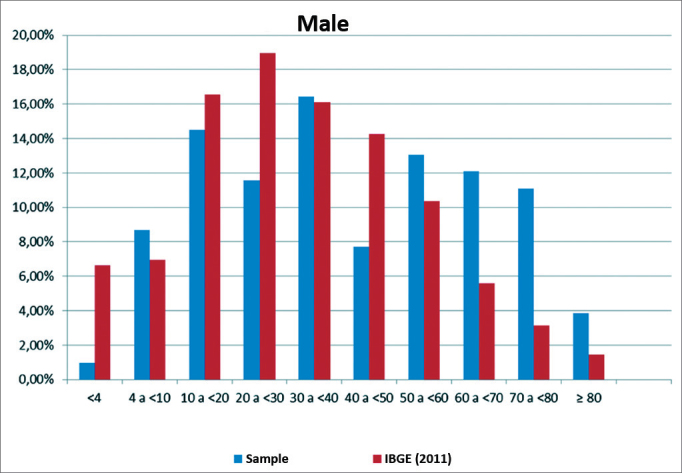
Comparison showing the distribution of the sample of male subjects per age group relative to population data (IBGE, 2011).

**Figure 2:**
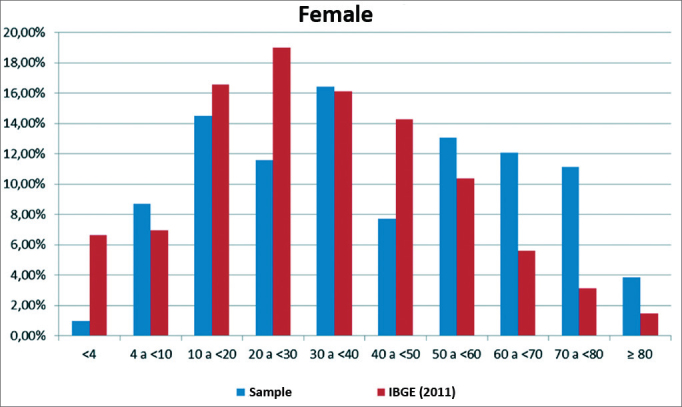
Comparison showing the distribution of the sample of female subjects per age group relative to population data (IBGE, 2011).

Figures 1 and 2 show a comparison between the sample distribution by sex and age group and population data (IBGE 2011) in Itajaí. These figures show more significant differences between sample and population (IBGE) in the age groups 0–4 years, 20–30, and over 70 years in male, and in the age groups 0–4 years, 40–50 years, and over 70 years in females.

**Figure 3:**
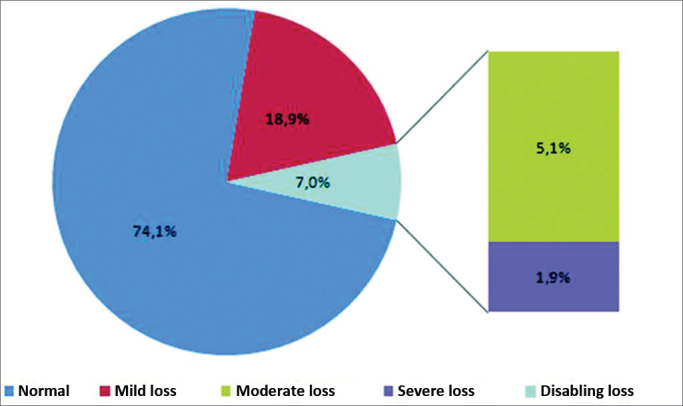
Percentage distribution of incapacitating hearing loss in subjects undergoing pure tone audiometry, Itajaí, SC, Brazil, 2011.

An analysis of the minimum hearing level in the best ear showed that 74.1% of subjects were normal-hearing, and 25.9% had some degree of hearing loss (18.9% – mild hearing loss, 5.1% – moderate hearing loss, 1.9% – severe hearing loss, 0% – profound hearing loss). Thus, the prevalence of incapacitating hearing loss was 7% (Figure 3).

Table 1 shows the prevalence of incapacitating hearing loss per age group; there were more cases in subjects aged over 50 years, and a predominance in subjects aged over 70 years.

**Table 1:** Prevalence of incapacitating hearing loss in subjects aged 4 years or more.

Hearing level in the best ear	Age group									
	4 to <10	10 to <20	20 to <30	30 to <40	40 to <50	50 to <60	60 to <70	70 to <80	≥ 80	General
Normal o mild loss	34	55	44	57	42	42	39	25	7	345
	97.14%	100.00%	100.00%	100.00%	97.67%	93.33%	92.86%	67.57%	53.85%	92.99%

Moderate or severe loss (incapacitating)	1	0	0	0	1	3	3	12	6	26
	2.86%	0.00%	0.00%	0.00%	2.33%	6.67%	7.14%	32.43%	46.15%	7.01%

Total	35	55	44	57	43	45	42	37	13	371

The mean noise in 137 complete households that were studied was 38.4 dB; the median was 38.3 dB, the minimum was 32.2 dB, and the maximum was 51.9 dB (standard deviation – 3.5). These numbers did not correlate with the presence or absence of hearing loss.

Tympanometry revealed a predominance of the type A curve (93.4% in the left ear and 93.1% in the right ear); there was a lower incidence of the type B curve (5.8% in the left ear and 5.3% in the right ear) e C (0.8% in the left ear and 1.7% in the right ear). All were conduction hearing loss cases. Similarly, acoustic reflexes were found in most of the study population. Considering all degrees of hearing loss, the type A curve and reflexes were the most frequent.

Relating personal and family health history and the presence of hearing/otologic complaints to incapacitating hearing loss – detected in 26 subjects of the sample – nine had no complaints, and the other 17 subjects had a positive history and hearing/otologic complaints, of which seven had associated symptoms (two manifested vertigo and five had tinnitus). Co-morbidities included diabetes mellitus (2 cases), systemic elevated arterial blood pressure (2 cases), a history of stroke (2 cases), and one subject had undergone chemotherapy. Of these 26 cases, one was a child (a diagnosis of otitis media with effusion, dysfunctional Eustachian tube, and adenoid hyperplasia – this child was referred for surgery); four were adults aged over 45 years (one case of chronic otitis media and past auditory surgery, one case apparently with noise induced hearing loss, and two of idiopathic causes, all of which were referred for fitting hearing aids). Among 21 elderly subjects with presbyacusis, 17 perceived their hearing loss (HHIE-S: 3 – mild-moderate, 14 – severe) and four had no perception of hearing loss. The HHIE-S of all 92 sample subjects concluded that 71 elderly subjects were classified as not having incapacitating hearing loss, of which 39 had no perception of hearing loss, 18 had mild to moderate perception, and 14 had severe perception. Of 21 elderly subjects with incapacitating hearing loss, one used a hearing aid bilaterally and was a patient of Practice Vale do Itajai University (UNIVALI), two refused to continue treatment, and 18 were referred for fitting of hearing aids. At present hearing aids have been fitted on 16 patients, and are being selected for two other patients. Considering all degree of hearing loss and including the hearing level of the worse ear, besides the 26 subjects with incapacitating hearing loss, another 109 subjects also had abnormal tests. For all 315 cases, the most frequent etiologies were: presbyacusis (40.74%), idiopathic (17.04%), ear wax (6.66%), chronic otitis media (5.92%), otosclerosis (3.70%), noise-induced hearing loss (2.22%), labyrinthic disease (1.48%), papilloma of the outer ear canal (0.74%), and associated causes (21.5%) (see Table 2). Of 109 non-incapacitating cases but with detected abnormalities, 54 are being monitored periodically for hearing, 53 were treated medically and successfully (drugs/lavage), one was referred for surgery (adenoidectomy and tympanotomy for placing a bilateral ventilation tube), and one has already been operated (removal of an outer ear canal papilloma). In the group of children aged below 4 years, all had the cochlea-palpebral reflex, three had normal tests, OAE were absent bilaterally in two cases, OAE were absent to the right in one cases, and OAE were absent to the left in one case. These abnormalities correlated with type B curves on tympanometry and absence of acoustic reflexes; there results normalized after medical evaluation and treatment.

**Table 2:** Likely etiology (all degrees of hearing loss).

Most likely etiology	Frequency 55	Percentage
Presbyacusis	55	40.74
Idiopathic	23	17.04
Presbyacusis+OME^a^+Dysfunctional ET^b^	11	8.14
Ear wax	9	6.66
COM^c^	8	5.92
Presbyacusis+COM	7	5.20
OME+Disfunctional ET	7	5.20
Otosclerosis	5	3.70
Presbyacusis+NIHL^d^	3	2.22
NIHL	3	2.22
Labyrinth disease	2	1.48
Idiopathic+Dysfunctional ET	1	0.74
Outer ear canal papilloma	1	0.74

Total	135	100.0

a otitis media with effusion;

b Eustachian tube;

c chronic otitis media;

d noise-induced hearing loss.

## DISCUSSION

This is the third study on a population survey about the prevalence of hearing loss after Béria et al.'s^6^ and Bevilacqua et al.'s^7^ papers; it was rigorously based on the WHO protocol, and is the first study to also take into account the etiology of hearing loss, thereby attempting to make the diagnosis, follow-up, and therapy.

The study sample consisted of male and female subjects, allocated to age groups ranging from less than 4 years to 80 years or more. The total sample comprised 379 evaluated subjects in 137 complete households, residents of 14 census sectors in Itajaí. The IBGE census data were applied to characterize and analyze the representativeness of households and groups of individuals; discrepancies were due to the fact that the IBGE employs more teams, interviewees are required to respond, t interviewers do not enter the households, that dwellers are not required to be in the house, and that the time taken for interviews is shorter. In our study, household dwellers had to agree and all had to be present in the house; it was also necessary to enter the households to carry out clinical interviews, which required more time. The most significant variations between the study sample and the IBGE census were found in the 0–4, the 20–30, and the 60+ age groups in both sexes and in the 40–50 years group in females. A smaller proportion of children aged up to 4 years, of subjects aged 20–30 years and 40–50 years in our sample is probably due to young children staying in crèches and nurseries and adults at work; older people predominated in the sample, reflecting the fact that this age groups stays more at home.

Visitation was made of 774 households, in which 715 people were evaluated; as the WHO criteria were followed strictly, these numbers were reduced to 137 complete households and 379 subjects in the sample. The most significant difficulties were due to the field survey. Although a letter was sent to the houses that were chosen to provide information about the project and letting the household dwellers know that a visit would be made by an Auditory Health team – a strategy that minimized refusal – many houses could not be included because the head of the family refused to participate. Another difficulty were the floods, which affected about 90% of the town in the second semesters of 2008 and 2009; this often made it impossible for the team to visit houses. Households in which all dwellers could not be evaluated were excluded from the sample; this was due to refusal to participate, absence in up to three visits, or rains and floods that resulted in destruction of houses and removal of household dwellers with subsequent loss of contact.

Most of the sample subjects were normal hearing, followed by cases with mild hearing loss (18.9%), as also noted by Béria et al.^6^ (19.3%) and Bevilacqua et al.^7^ (11.7%). Profound hearing loss was not found, again as in Bevilacqua et al.'s paper^7^.

The distribution of hearing loss per census sectors revealed no predominance of incapacitating hearing loss in specific census sectors.

The mean intensity of household noise and the number of dwellers per household did not influence the presence or not of incapacitating hearing loss.

The prevalence of incapacitating hearing loss in Itajaí was 7%; this is similar to the value Béria et al.^6^ found in Canoas, RS (6.8%). Our result differed from the value Bevilacqua et al.^7^ found in Monte Negro, RO (4.8% prevalence of incapacitating hearing loss). Because these towns/cities are located in different parts of the country (North and South regions), the above-mentioned differences may be due to local factors, raising issues and hypotheses that may be investigated in other studies.

Most cases of incapacitating hearing loss were found in individuals aged over 50 years; it predominated in individuals aged over 70 years. This results is similar to that published by Béria et al.^6^ and Bevilacqua et al.;^7^ both authors encountered a prevalence of incapacitating hearing loss at ages respectively over 60 years and 50 years. Our results indicate that it is important to take into account the age groups of subjects when establishing the prevalence of hearing loss, and to compare the results of several municipalities.

The personal health history and the auditory complaints showed that 282 of 345 subjects without incapacitating hearing loss had no complaints; however, 63 subjects without incapacitating hearing loss did have complaints about hearing. Of 26 sample subjects that had incapacitating hearing loss, 17 had complaints and nine were complaint-free. The HHIE-S of 92 elderly subjects in the ample showed that 39 of 71 elderly individuals classified as not having incapacitating hearing loss had no perception of hearing loss; 18 had mild to moderate perception, and 14 had severe perception. In the group of 21 elderly subjects with incapacitating hearing loss, 17 had perception of hearing loss, three had a HHIE-S showing mild to moderate perception, 14 had severe perception, and four did not have any perception of loss. Auditory complaints and/or perception of hearing loss may be – in the study sample – considered indicative factors of the presence of incapacitating hearing loss.

Even in cases not classified as incapacitating (mild loss, unilateral loss or asymmetric loss), many subjects also had complaints and perception of incapacity, showing the negative impact of hearing loss, irrespective of its degree, on the quality of life of individuals with hearing problems.

Another interesting result in our study was that when all detected abnormalities and the levels of the worse ear were taken into account, 109 subjects (28.76%) presented abnormalities, of which about half (55 cases – 50.46%) were solved by medical and/or surgical therapy, and another half (54 cases – 49.54%) were asked to return for follow-up of their auditory or otologic condition. Of the 26 incapacitating cases of hearing loss, 24 (92.31%) have already been or are being rehabilitated; one individual already used a hearing aid, was a patient of the ‘Hearing Health Care Unit' and a member of the municipality, one patient successfully underwent otorhinolaryngologic surgery, and two refused to return for follow-up.

The most frequent etiologies of hearing loss among the population with incapacitating hearing loss in our sample were: presbyacusis(80.76%), chronic otitis media (7.69%), idiopathic conditions (7.69%), and noise-induced hearing loss (3.84%); thus, 11.53% were potentially avoidable.

These data underline the importance of early detection and treatment of hearing loss, as most may be resolved or improved with therapy.

## CONCLUSION

The prevalence of incapacitating hearing loss (moderate, severe, and profound) in Itajaí was 7%; it predominated in individuals aged over 50 years, and the main cause was presbyacusis.

At the same time, it was clear that by detecting hearing problems in this specific population, treatments and procedures were undertaken that solved and/or minimized the hearing losses that were encountered.

It is hoped that this study may contribute to developing a database with information about hearing in Itajaí, and that in future this initiative may be extended to other municipalities in the state of Santa Catarina. This database may guide measures, minimize costs, optimizing and directing funds for the best use in assessing, diagnosing, and rehabilitating individuals with hearing loss.
